# Hardware-Oriented Approximations of Softmax and RMSNorm for Efficient Transformer Inference

**DOI:** 10.3390/mi17010084

**Published:** 2026-01-07

**Authors:** Yiwen Kang, Dong Wang

**Affiliations:** 1 Institute of Information Science, Beijing Jiaotong University, Beijing 100044, China; yiwenkang@bjtu.edu.cn; 2 Beijing Key Laboratory of Advanced Information Science and Network Technology, Beijing 100044, China

**Keywords:** transformer inference, hardware acceleration, FPGA, Softmax, RMSNorm

## Abstract

With the rapid advancement of Transformer-based large language models (LLMs), these models have found widespread applications in industrial domains such as code generation and non-functional requirement (NFR) classification in software engineering. However, recent research has primarily focused on optimizing linear matrix operations, while nonlinear operators remain relatively underexplored. This paper proposes hardware-efficient approximation and acceleration methods for the Softmax and RMSNorm operators to reduce resource cost and accelerate Transformer inference while maintaining model accuracy. For the Softmax operator, an additional range reduction based on the SafeSoftmax technique enables the adoption of a bipartite lookup table (LUT) approximation and acceleration. The bit-width configuration is optimized through Pareto frontier analysis to balance precision and hardware cost, and an error compensation mechanism is further applied to preserve numerical accuracy. The division is reformulated as a logarithmic subtraction implemented with a small LOD-driven lookup table, eliminating expensive dividers. For RMSNorm, LOD is further leveraged to decompose the reciprocal square root into mantissa and exponent parts, enabling parallel table lookup and a single multiplication. Based on these optimizations, an FPGA-based pipelined accelerator is implemented, achieving low operator-level latency and power consumption with significantly reduced hardware resource usage while preserving model accuracy.

## 1. Introduction

Since the Transformer model was proposed in 2017 [1], it has demonstrated strong capabilities in parallel sequence processing through global attention modeling and has become the dominant architecture for large language models (LLMs). In recent years, the Transformer architecture has continued to evolve, and the emergence of open-source models such as OPT, LLaMA, LLaMA2 [2,3,4], and LLaMA3 has made the private deployment of LLMs increasingly feasible. Despite these advances, the parameter scale of LLMs has increased extremely rapidly in recent years, while hardware performance—constrained by Moore’s Law—has improved by only about 1.5× every 18 months [5]. This imbalance introduces significant challenges in storage, computation, and power consumption, particularly for inference on edge devices where energy budgets are extremely limited. As a result, it hinders the widespread adoption of LLMs in real-world applications. Field Programmable Gate Arrays (FPGAs), with their inherent low-power characteristics and reconfigurable computing resources, offer a promising platform for energy-efficient and flexible inference acceleration in embedded and edge systems. Consequently, operator-level optimization of Transformer inference on FPGA and other embedded platforms has become an active research direction [6,7,8,9].

Deploying Transformer models on FPGAs presents significant challenges due to the high computational cost of nonlinear operations. While linear layers benefit from low-bit quantization and specialized matrix–vector multiplication (MVM) engines, key nonlinear functions such as Softmax and RMSNorm still rely on high-precision floating-point arithmetic. Beyond conventional digital accelerators, analog and mixed-signal in-memory computing has been explored to speed up large-scale MVM in neural networks [10,11]. However, these approaches mainly target linear operations and do not address the FPGA challenges of nonlinear operators.

As a result, nonlinear functions such as Softmax and RMSNorm introduce considerable latency and resource overhead. This effect is particularly pronounced for long-context inference (e.g., 4096 tokens), where these operators can account for a significant fraction of the total inference runtime [12]. Many existing accelerator designs provide limited implementation details or rely on floating-point arithmetic and dynamic-shift operations [9,13,14], which are inefficient on FPGAs.

To address these challenges, this work presents hardware-oriented algorithmic designs for the two key nonlinear operators—Softmax and RMSNorm and implements them on an FPGA platform. The main contributions are summarized as follows:

- Softmax Approximation and Acceleration: The exponential function is approximated over a wide input range using lookup tables (LUTs) and a single adder, enabling an efficient, fully fixed-point implementation without requiring any DSP resources.
- RMSNorm Approximation and Acceleration: The reciprocal square root function $x^{-0.5}$ is approximated using an LOD–LUT–MUL structure, where exponent and mantissa components are independently processed by 32- and 256-entry LUTs, respectively. Their outputs are multiplied to reconstruct $x^{-0.5}$, enabling a fully fixed-point realization with minimal DSP usage while preserving numerical precision.
- Hardware Implementation and Evaluation: A deeply pipelined fixed-point architecture is designed for both operators and deployed on the Xilinx Alveo U55C accelerator card, achieving low resource utilization, reduced inference latency, and competitive precision.

## 2. Related Work

As large language models (LLMs) are rapidly expanding into industrial applications, including operation log analysis, real-time production monitoring, and QA systems, there is an urgent need for hardware architectures that can execute Transformer operators with low resources and power consumption.

Numerous studies have explored hardware-friendly approximations and accelerators of the Softmax function to enhance Transformer inference efficiency. Softermax [15] replaces the exponential function with a base-2 power form and introduces Online-Softmax to reduce the number of iterations. Although this approach improves computational efficiency, it introduces an additional exponential computation and requires fine-tuning to recover precision loss, thereby limiting its practicality on FPGA platforms. Mei [16] applies a three-segment PLAC (Piecewise Linear Approximation Calculation) method for exponential approximation, where each segment requires an independent multiplier, leading to considerable hardware cost. Koca [17] expresses the exponential function as $e^{x}\approx 2^{1.5x}$, where the constant $\log_{2}e\approx 1.5$ replaces the multiplication factor, allowing the use of shift operations instead of costly multipliers. However, the method relies on dynamic shifters to scale the exponential output, which incurs substantial hardware overhead on FPGA devices. Wang [18] proposes SOLE, which employs power-of-two scaling to approximate $e^{x}$ as $2^{kx}$ and replaces the normalization term with its nearest power of two, thereby eliminating division operations and significantly reducing hardware cost. Nevertheless, the method has not been evaluated on FPGA platforms. Overall, these approaches alleviate the Softmax bottleneck to varying degrees. However, most are not tailored to FPGA architectures or fail to meet the high-precision requirements of attention.

Compared with Softmax and LayerNorm, hardware optimization studies on RMSNorm remain relatively limited. RMSNorm removes mean subtraction, which reduces data dependency and makes it more hardware friendly. However, it still requires squaring, accumulation, and reciprocal square root. Lu [19] computes reciprocal square root using direct arithmetic. Yu [20] uses piecewise polynomials but incurs high cost under a wide dynamic range. Kim [12] uses Newton–Raphson iteration, which needs extra multipliers and adders and is less suitable for resource-constrained FPGAs. The SOLE architecture [18] quantizes the $x^{2}$ operation to a lower bit-width representation to reduce complexity, but it depends on a quantization scheme poorly suited for models with large parameter scales. Li [21] proposes a general division approximation technique. However, its direct extension to LayerNorm results in excessive lookup-table overhead. In general, dedicated hardware designs for RMSNorm remain largely unexplored. Overall, dedicated RMSNorm hardware designs remain largely unexplored.

Compared to the SOLE [18] method, which relies on a resource-intensive dynamic shifter, our Softmax approximation adopts a LUT-based scheme. By transforming division into the log domain, we reuse the same exp-LUT and eliminate the need for a dedicated divider. Compared to Li’s LayerNorm approximation [21], which extends general division to square-root division but overlooks the sign and parity of *k* in $1/\sqrt{x}=2^{-k/2}\left(1+s\right)$, requiring two large LUTs and four processing branches. Our design replaces these with only two small LUTs and a multiplier, simplifying structure while preserving accuracy. Compared to HAAN [22], our reciprocal–root scheme stays fully in the fixed-point domain and avoids costly FX–FP–FX conversions, reducing logic and DSP usage. Compared to Kim [12], which supports a narrow input range (0, 2), our method extends the valid range, which is more suitable for LLM workloads.

## 3. Preliminary

### 3.1. Softmax and RMSNorm

In the Transformer architecture, nonlinear normalization and activation functions are essential for stable training and numerical robustness. Among them, Softmax and RMSNorm are core operations in the self-attention and residual normalization modules, respectively. Softmax converts the similarity scores between Query and Key into normalized attention weights. Given the attention vector $x=[x_{1},x_{2},\ldots ,x_{n}]$, it is defined as:

(1) $$\mathrm{Softmax}\left(x_{i}\right)=\frac{e^{x_{i}}}{\sum_{j=1}^{n}e^{x_{j}}}.$$

To prevent numerical overflow, a stabilized version is typically adopted [23,24]:

(2) $$\mathrm{SafeSoftmax}\left(x_{i}\right)=\frac{e^{x_{i}-\max\left(x\right)}}{\sum_{j=1}^{n}e^{x_{j}-\max\left(x\right)}}.$$

Its main challenge arises from the exponential and division operations, which introduce significant computational complexity and hardware overhead in float implementations.

RMSNorm is a simplified variant of LayerNorm that removes the mean subtraction step, and then normalizes using the root mean square (RMS) of the input vector. Given $x=[x_{1},x_{2},\ldots ,x_{d}]$, RMSNorm is defined as:

(3) $$\mathrm{RMSNorm}\left(x\right)=\frac{x}{\sqrt{\frac{1}{d}\sum_{i=1}^{d}x_{i}^{2}+\epsilon }},$$

where *d* is the input dimension, ϵ is a small constant for numerical stability. By eliminating the mean subtraction term $\left(x_{i}-\bar{x}\right)$ of Layernorm, RMSNorm reduces computation and intermediate storage, making it more hardware-friendly, particularly for low-latency and resource-constrained devices. Its main challenge lies in the reciprocal square root operation, which remains expensive in hardware, motivating further research into efficient approximation and accelerator methods.

### 3.2. Bipartite-Table

**Figure 1:**
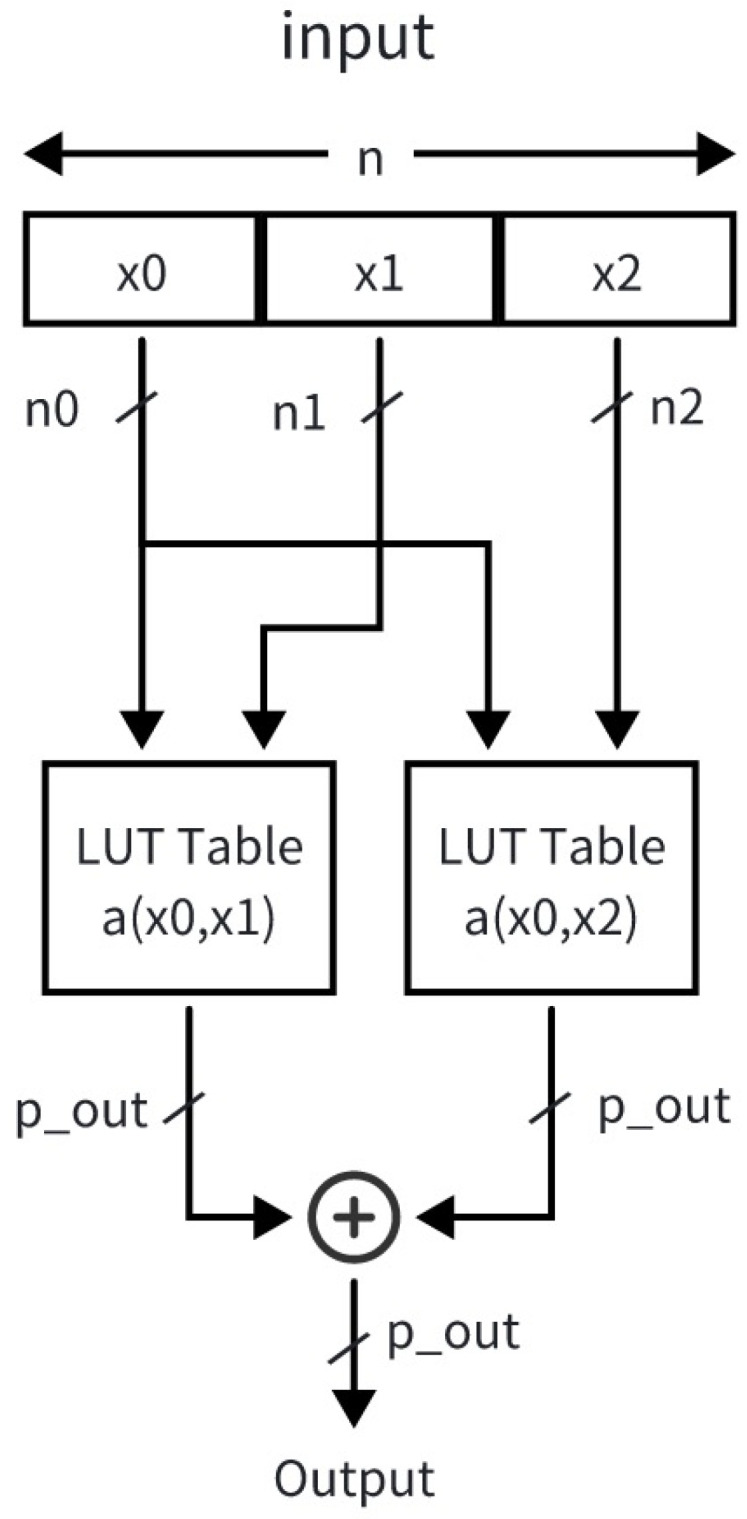
The bipartite table method. The method partitions the input and performs parallel lookup for high-precision approximation accelerator.

The Bipartite Table [25] method is a lookup-based algorithm for high-precision function approximation. Figure 1 illustrates its structure. The method partitions the input into multiple segments. It performs parallel table lookups. This design reduces storage requirements and preserves high computational accuracy. The basic idea is as follows: given an input operand *x*, it is divided into three parts $x_{0},x_{1},x_{2}$ with bit widths $n_{0},n_{1},n_{2}$, respectively, such that the target function f(x) can be approximated by a two-term expansion:

(4) $$f\left(x\right)\approx a_{0}\left(x_{0},x_{1}\right)+a_{1}\left(x_{0},x_{2}\right).$$

Here, the first term $a_{0}\left(x_{0},x_{1}\right)$ is generated by the $n_{0}+n_{1}$ bits (the $x_{0}$ and $x_{1}$ segments) and accessed from the first lookup table (Table $a_{0})$ to provide the main approximation value, while the second term $a_{1}\left(x_{0},x_{2}\right)$ uses the $n_{0}+n_{2}$ bits segments as input to the second table (Table $a_{1}$) to generate a local correction term.

The outputs of the two tables are then summed to produce the final approximation result. The Bipartite Table method is hardware-friendly because it replaces a single large lookup table with two much smaller ones, significantly reducing the number of stored entries while maintaining high-precision function approximation.

### 3.3. Compensation-Based Approximation Principle

To further reduce the accelerator’s approximation bias introduced by table-based nonlinear function estimation, a compensation mechanism was introduced in prior work [21]. The key idea is that when a continuous function f(x) is uniformly quantized into discrete segments, the value retrieved from the lookup table $\tilde{f}\left(x\right)$ may deviate from the true mean of f(x) over that interval. To correct this systematic bias, the average deviation between f(x) and its quantized representation is precomputed for each segment and stored as a small compensation table. During inference, the corresponding compensation value is subtracted from the lookup output, yielding a bias-corrected approximation as:

(5) $$f_{\mathrm{corr}}\left(x\right)=\tilde{f}\left(x\right)-\Delta_{k},\;\Delta_{k}=\frac{1}{\Delta x}\;\int_{x_{k}}^{x_{k+1}}\left(\tilde{f}\left(x_{k}\right)-f\left(x\right)\right)\;dx.$$

This formulation can be applied generically to functions such as the reciprocal and square-root operations, providing a simple yet effective way to enhance accelerator approximation accuracy without increasing table resolution or computational complexity. In this work, we adopt and refine this decomposition strategy for RMSNorm.

## 4. Methods

### 4.1. Range-Reduced LUT-Based Approximation for Softmax

In the Softmax function, the exponential input spans from negative infinity to positive infinity, making direct hardware approximation infeasible for accelerator implementations. The SafeSoftmax technique mitigates this issue by subtracting the maximum element, constraining the input to (−∞,0] as introduced in Section 3.1. However, this range remains too wide for lookup-table (LUT) implementation.

By examining the numerical magnitude of the exponential term under fixed-point inference, we observe that when x<−16, the exponential value satisfies $e^{x}\leq e^{-16}\approx 1.1\times 10^{-7}$, which is negligible compared with the dominant terms in the Softmax denominator. In our experiments on LLaMA2-7B inference, truncating exponential inputs below this threshold results in only a very small and practically negligible increase in perplexity. Similar exponential range truncation strategies have also been adopted in prior works [26]. Therefore, in this work, we empirically restrict the effective exponential input range to (−16,0] to simplify FPGA implementation while preserving numerical accuracy.

Within this bounded range, the Bipartite Table method described in Section 3.2 is employed to approximate the exponential efficiently. Since all input terms $\left(x_{i}-\max\left(x\right)\right)$ are non-positive, the index is redefined as $(\max\left(x\right)-x_{i})\in \left[0,16\right)$, avoiding additional sign-bit processing. Because the clipped input range is [0, 16), its integer part can be represented using only 4 bits. As shown in Table 1, experiments indicate that allocating 7 bits for intput fractional part is sufficient to maintain accuracy. The output bit width is determined by referring to the settings in [21] and combined with experimental results. 15 fractional bits are required to represent the self-attention scores in the range [0, 1] with sufficient precision. Ultimately, using an 11-bit input (4 integer bits and 7 fractional bits) and a 16-bit output (1 integer bit and 15 fractional bits) achieves accuracy comparable to floating-point computation while maintaining hardware efficiency.

The input variable x∈[0,16) is decomposed into three non-overlapping parts:

(6) $$x=x_{0}+x_{1}+x_{2},$$

where each segment corresponds to distinct bit widths. The Bipartite Table method then approximates the exponential function using two compact LUTs and one adder, achieving high precision with minimal memory overhead.

### 4.2. Bipartite LUT Configuration and Error Compensation

In the Bipartite Table method [25], the input *x* is divided into three parts $x_{0},x_{1},x_{2}$ with bit widths $n_{0},n_{1},n_{2}$, respectively. The total bit width is $n=n_{0}+n_{1}+n_{2}$. Increasing precision in one segment exponentially enlarges the table size, leading to higher storage and latency. Therefore, determining an appropriate bit-width allocation is essential to balance precision and resource utilization.

#### 4.2.1. Two-Stage Bit-Width Search via SNDR Analysis and Pareto Pruning

To balance approximation accuracy and hardware storage cost, we adopt a two-stage configuration exploration strategy. In the first stage, an analytical error model based on the signal-to-noise-and-distortion ratio (SNDR) is developed to eliminate inefficient bit-width configurations. In these configurations, the overall SNDR is dominated by a single error source, such that increasing other bit widths no longer leads to meaningful accuracy improvement. In the second stage, the remaining feasible configurations are further pruned by a Pareto frontier analysis in the accuracy-cost space to obtain a compact candidate set for empirical evaluation.

Following [25], the total approximation error is decomposed into three components: $E_{0}$, the truncation error from Taylor expansion; $E_{1}$, the coefficient quantization error; and $E_{2}$, the output quantization error. The overall SNDR is defined as

(7) $$\begin{array}{cc} \mathrm{SNDR} & =10\log_{10}\;\left(\frac{P_{s}}{P_{n}+P_{D}}\right)\\ \; & =10\log_{10}\;\left(\frac{f{\left(x\right)}^{2}}{E_{0}^{2}+E_{1}^{2}+E_{2}^{2}}\right), \end{array}$$

where $P_{s}$ denotes the signal power and $P_{n}+P_{D}$ represents the total noise and distortion power.

Assuming that the three error sources are independent and uniformly distributed, their magnitudes can be bounded as

(8) $$\begin{array}{cc} E_{0} & <\;|f^{\prime \prime }\left(\xi_{1}\right)|\;\cdot \;2^{-2n_{0}-2n_{1}-2}, \end{array}$$

(9) $$\begin{array}{cc} E_{1} & <\;|f^{\prime \prime }\left(\xi_{2}\right)|\;\cdot \;2^{-2n_{0}-n_{1}-2}, \end{array}$$

(10) $$\begin{array}{cc} E_{2} & \leq 2^{-n_{\mathrm{out}}}. \end{array}$$

Given the clipped input range [0,16), the average signal power can be approximated as

(11) $$P_{s}=E\left[e^{-2x}\right]=\frac{1}{16}\;\int_{0}^{16}e^{-2x}\;dx\approx \frac{1}{32}.$$

Substituting the error bounds yields

(12) $$\mathrm{SNDR}\approx 10\log_{10}\;\left(\frac{1/32}{2^{-4n_{0}-4n_{1}-4}+2^{-4n_{0}-2n_{1}-4}+2^{-2n_{\mathrm{out}}}}\right).$$

The SNDR expression shows that the total error is dominated by the largest of the three error sources. Once a single error term becomes dominant, reducing other errors provides little SNDR improvement. Therefore, bit-width allocation should aim to balance the major error sources.

Since the first error term $E_{0}$ is always smaller than $E_{1}$, the effective balance can be achieved by equating $E_{1}$ and $E_{2}$, which yields the bit-width relation:

(13) $$\begin{array}{c} n_{\mathrm{out}}\leq 2n_{0}+n_{1}+2, \end{array}$$

(14) $$\begin{array}{c} n_{in}=n_{0}+n_{1}+n_{2}. \end{array}$$

After applying the SNDR-based constraint, multiple configurations may still satisfy the condition while exhibiting significantly different hardware costs and approximation accuracies. Therefore, a second pruning step is introduced using a Pareto frontier analysis.

The hardware cost metric *C* is defined as the total number of LUT entries required by $a_{0}\left(x_{0},x_{1}\right)$ and $a_{1}\left(x_{0},x_{2}\right)$:

(15) $$C=2^{n_{0}+n_{1}}+2^{n_{0}+n_{2}}.$$

The accuracy metric *A* is defined based on the dominant error term $E_{1}\propto 2^{-(2n_{0}+n_{1})}$ after SNDR balancing:

(16) $$A=2n_{0}+n_{1},$$

In the (C,A) space, a configuration *i* is said to be dominated if there exists another configuration *j* such that

(17) $$C_{j}\leq C_{i}\;\mathrm{and}\;A_{j}\geq A_{i},$$

and at least one inequality is strict. Only non-dominated configurations on the Pareto frontier are retained for further experimental evaluation.

**Figure 2:**
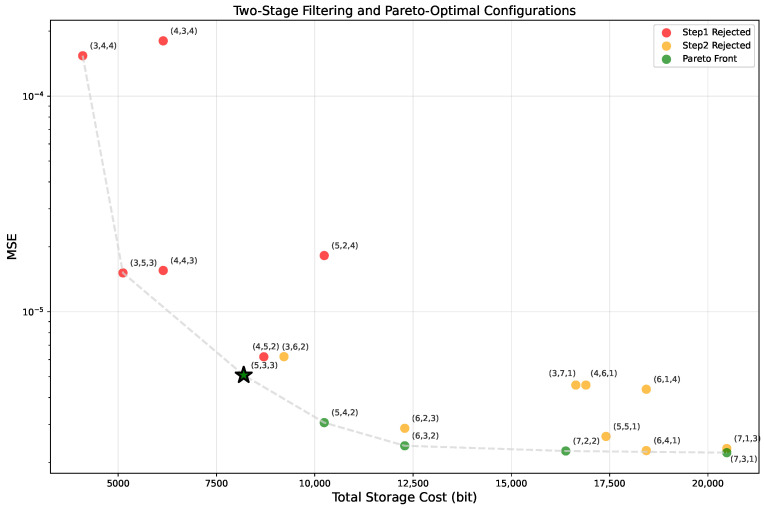
Two-Stage Filtering and Pareto-Optimal Configurations. The green five-pointed star highlights the selected configuration $\left(n_{0},n_{1},n_{2}\right)=\left(5,3,3\right)$.

As shown in Figure 2, the red points are filtered out in the first stage using the SNDR-based constraint in Equation (13), the orange points are further removed by the Pareto optimality criterion in Equation (17), and the remaining green points are selected for subsequent experimental evaluation.

As shown in Table 2, we select (5,3,3) as the final bit-width configuration for $\left(n_{0},n_{1},n_{2}\right)$. Accordingly, $n_{0}=5$ and $n_{1}=3$ are used to index the $a_{0}$ lookup table, while $n_{0}=5$ and $n_{2}=3$ are used to index the $a_{1}$ lookup table. In total, this configuration requires $2^{5+3}+2^{5+3}=512$ table entries.

#### 4.2.2. Error Compensation and LUT Optimization

Even with optimal bit-width allocation, the bipartite LUT structure still introduces residual quantization errors caused by input truncation, function approximation, and output rounding. To further enhance numerical accuracy, this work introduces a lightweight error compensation mechanism that fine-tunes the LUT coefficients through parameterized optimization.

Let the *n*-bit quantization operator be defined as

(18) $$Q_{n}\left(x\right)=2^{-n}\cdot \left\lfloor 2^{n}x\right\rceil ,$$

where ⌊·⌉ denotes rounding to the nearest integer. Based on this operator, the compensated LUT entries are expressed as:

(19) $$\begin{array}{cc} {\tilde{a}}_{0}\left(x_{0},x_{1}\right) & =2^{-n_{\mathrm{out}}}\;\left\lfloor 2^{n_{\mathrm{out}}}\exp\;\left(-Q_{n_{0}}\left(x_{0}\right)-Q_{n_{1}}\left(x_{1}\right)-\delta_{2}\right)\right\rceil , \end{array}$$

(20) $$\begin{array}{cc} {\tilde{a}}_{1}\left(x_{0},x_{2}\right) & =2^{-n_{\mathrm{out}}}\;\left\lfloor 2^{n_{\mathrm{out}}}\left[-\;\exp\;\left(-Q_{n_{0}}\left(x_{0}\right)-\delta_{1}-\delta_{2}\right)\left(Q_{n_{2}}\left(x_{2}\right)-\delta_{2}\right)\right]\right\rceil , \end{array}$$

where $\delta_{1}$ and $\delta_{2}$ are exactly halfway between the minimum and maximum values for x1 and $x_{2}$:

(21) $$\delta_{1}=2^{-(n_{0}+1)}-2^{-(n_{0}+n_{1}+1)},\;\delta_{2}=2^{-(n_{0}+n_{1}+1)}-2^{-(n_{0}+n_{1}+n_{2}+1)}.$$

The final exponential approximation is then given by

(22) $$\hat{f}\left(x\right)={\tilde{a}}_{0}\left(x_{0},x_{1}\right)+{\tilde{a}}_{1}\left(x_{0},x_{2}\right).$$

To jointly minimize mean squared error (MSE) and maximum absolute error (MAX) approximation errors, a hybrid loss function is employed.

(23) $$L\left(f,\hat{f}\right)=\alpha \;\frac{1}{N}\sum_{i=1}^{N}\;{\left(f\left(x_{i}\right)-\hat{f}\left(x_{i}\right)\right)}^{2}+\beta \;\max_{1\leq i\leq N}\;|f\left(x_{i}\right)-\hat{f}\left(x_{i}\right)|,$$

where α,β>0 are weighting coefficients. This optimization ensures smooth convergence of LUT coefficients and numerical stability in hardware deployment.

The final compensated exponential can thus be efficiently evaluated as:

(24) $$e^{x_{i}-\max\left(x\right)}\approx {\mathrm{LUT}}_{a_{0}}\;\left[\max\left(x\right)-x_{i}\right]+{\mathrm{LUT}}_{a_{1}}\;\left[\max\left(x\right)-x_{i}\right],$$

Near–floating-point precision is achieved with minimal hardware cost.

#### 4.2.3. Training and Experimental Setup

This section details the error compensation training procedure, parameter settings, and evaluation results. The training data cover the range [0,16], corresponding to the absolute value of the exponent input after clipping in the Softmax function, and consist of 10,000 uniformly sampled points. A separate test set of 1,000,000 randomly generated samples is used for final error evaluation.

The weighting coefficients in the hybrid loss are set to α=0.5 and β=1.0, determined via the best trade-off between mean squared error and maximum absolute error after several tries. Optimization is performed in PyTorch 3.9.23 using the Adam optimizer with a learning rate of $10^{-4}$ for 1000 epochs.

The trainable lookup table (LUT) entries $a_{0}$ and $a_{1}$ are initialized using the analytical expressions provided in Equations (19) and (20), respectively. The parameters $n_{0}$, $n_{1}$, and $n_{2}$ are set to the Pareto-optimal configuration (5,3,3), as determined in the previous Section 4.2.1. During training, fake quantization is applied in the forward pass. The Straight-Through Estimator (STE) is then employed to approximate the gradient of the rounding operation during backpropagation.

**Figure 3:**
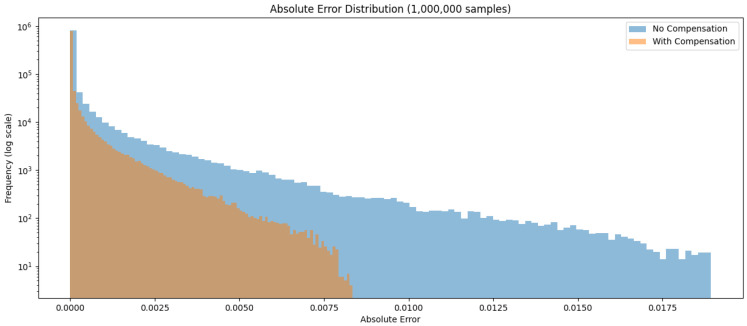
Comparison of absolute error distributions before and after compensation.

The absolute error distribution evaluated on a test set of 1,000,000 samples is visualized in Figure 3.

We compare three LUT-based exponential approximation methods: (1) Naive-lut, which uses a single LUT over the range [0,16]; (2) Bi-lut, the uncompensated dual-table approach; and (3) Bi-lut-comp, the compensated dual-table method. The comparison results are summarized in Table 3. The compensated Bipartite method (Bi-lut-comp) reduces the maximum absolute error by approximately 50% compared to the uncompensated version, demonstrating the effectiveness of the gradient-based compensation. Across all error metrics, Bi-lut-comp consistently outperforms Bi-lut-uncomp, while requiring only one-fourth of the LUT entries of Naive-lut. Although its absolute error remains slightly higher than that of Naive-lut (approximately twice), the resulting perplexity (PPL) degradation remains within an acceptable range.

### 4.3. Low-Cost Division Approximation for Softmax via Log-Domain Transformation

Inspired by previous work [27] on reformulating the Softmax function, we propose a division-free implementation based on lookup tables. Starting from the conventional SafeSoftmax expression, the function can be written as:

(25) $$f\left(x_{i}\right)=\frac{e^{-x_{i}}}{\sum_{j=1}^{N}e^{-x_{j}}},\;i=1,2,\ldots ,N.$$

To avoid expensive division operations, the exponential-to-logarithmic transformation is adopted:

(26) $$\begin{array}{cc} f(x_{i}) & =e^{\ln\;f(x_{i})}\\ \; & =\exp(-x_{i}-\ln\left(\sum_{j=1}^{N}e^{-x_{j}}\right))\\ \; & =\exp(-\left(x_{i}+\ln\left(\sum_{j=1}^{N}e^{-x_{j}}\right)\right)). \end{array}$$

The term $\ln(\sum e^{-x_{j}})$ can be efficiently approximated using a leading-one detector (LOD) and small lookup tables, while the outer exponential is handled by reusing the inner lookup table, as illustrated in the following subsections.

#### 4.3.1. Efficient Approximation of the Logarithmic Sum

Given $Sum=\sum e^{x_{j}}$, the LOD module detects the most significant bit of Sum in its binary form, decomposing it as:

(27) $$Sum=2^{k}\cdot \left(1+s\right),$$

where *k* is an integer and s∈[0,1) is the normalized mantissa. Thus,

(28) ln(Sum)=k·ln(2)+ln(1+s).

After SafeSoftmax processing, all input terms satisfy $x_{j}\leq 0$, yielding 1≤Sum<N. Thus, $k\in [0,\log_{2}N)$, and only $\left\lceil \log_{2}\left(\log_{2}N\right)\right\rceil$ bits are required to represent its integer part. The fractional part *s* is quantized to several bits (e.g., 4–8 bits), and its logarithm can be efficiently retrieved from a small LUT.

Truncating *s* to $b_{s}$ fractional bits introduces a bounded error:

(29) $$\delta =\ln S-\ln\tilde{S}=\ln\left(1+s\right)-\ln\;\left(1+T(s)\right)\geq 0,$$

where $S=\sum e^{x_{j}}$, $\tilde{S}=2^{k}\;\left(1+T(s)\right)$, and $T\left(s\right)=\left\lfloor s2^{b_{s}}\right\rfloor /2^{b_{s}}$. Consequently, the overall Softmax output is scaled by $\alpha =e^{\delta }$ with a one-sided bound:

(30) $$\begin{array}{cc} \tilde{\mathrm{Softmax}}\left(x_{i}\right) & =e^{-(x_{i}+\ln\tilde{S})}=e^{-(x_{i}+\ln S-\delta )}=\mathrm{Softmax}\left(x_{i}\right)\;e^{\delta },\\ \; & e^{\delta }\in \left[e^{-2^{-b_{s}-1}},\;e^{-2^{-b_{s}}}\right]\approx 1+O\left(2^{-b_{s}}\right). \end{array}$$

It was observed that truncating s to 4 bits results in a scaling variation of approximately 3–6%. This variation is tolerable for large-scale transformer models because RMSNorm following Softmax exhibits scale invariance. For $z\in R^{d}$ and α>0, the scaling property of RMSNorm can be expressed as:

(31) $${\mathrm{RMSNorm}}_{\epsilon }\left(\alpha z\right)=\gamma \;\frac{\alpha z}{\sqrt{\alpha^{2}{∥z∥}_{2}^{2}/d+\epsilon }}\approx {\mathrm{RMSNorm}}_{\epsilon }\left(z\right),\;\mathrm{when}\;\epsilon \ll {∥z∥}_{2}^{2}/d.$$

Thus, RMSNorm is exactly scale-invariant when ϵ=0, and approximately invariant when $\epsilon \ll {∥z∥}_{2}^{2}/d$ [28].

To verify the scale-invariance property in Equation (31) under practical conditions, we analyze the relative magnitude of the ϵ term and the relative error of RMSNorm outputs with scaled input. On Wikitext-2, the RMSNorm ϵ is set to $10^{-6}$, and the scaling factor is set to the worst case α=1.06 corresponding to a 6% scaling error caused by Softmax approximation. The experiment shows that the median (P50) value of $\epsilon /({∥z∥}_{2}^{2}/d)$ is $3.1\times 10^{-2}$, and the 95th percentile is below $1.6\times 10^{-1}$. This indicates that, for most tokens, the normalization term ${∥z∥}_{2}^{2}/d$ dominates ϵ, and RMSNorm therefore operates in an approximately scale-invariant regime. A small fraction of tokens exhibits larger ratios. Since ϵ is $10^{-6}$, the corresponding ${∥z∥}_{2}^{2}/d$ values are small as well, indicating that the hidden-state activations are very small in magnitude. Therefore, even when these activations are scaled by α=1.06 due to the Softmax approximation, their impact on subsequent computations remains limited.

To directly quantify the effect of input scaling on RMSNorm outputs, we measure the relative $\ell_{2}$ deviation between RMSNorm outputs before and after scaling. While the Softmax approximation introduces a 6% relative scaling perturbation at the input, the resulting RMSNorm output deviation is much smaller: the mean relative $\ell_{2}$ deviation is 0.3%, and the 95th percentile remains below 0.78%. This demonstrates that RMSNorm effectively suppresses the input scaling error in practice.

#### 4.3.2. Reuse of Exponential Unit

To further reduce hardware cost, the inner-stage exponential $e^{x_{\mathrm{inner}}}=e^{x_{i}-\max\left(x\right)}$ and the outer-stage exponential $e^{x_{\mathrm{outer}}}=e^{\left(x_{i}-\max\left(x\right)\right)-\ln\left(\sum_{j}e^{x_{j}-\max\left(x\right)}\right)}$ share the same lookup-table based exponential unit. First, since both $x_{\mathrm{inner}}\leq 0$ and $x_{\mathrm{outer}}\leq 0$, the outputs of $e^{x_{\mathrm{inner}}}$ and $e^{x_{\mathrm{outer}}}$ both lie in the range (0,1], allowing a unified LUT design for exponential approximation. Second, the input of the outer-stage exponential can be expressed as

(32) $$\begin{array}{cc} x_{\mathrm{outer}}\; & =\left(x_{i}-\max\left(x\right)\right)-\ln\;\left(\sum e^{x_{j}-\max\left(x\right)}\right)\\ \; & =x_{\mathrm{inner}}-\ln\;\left(\sum e^{x_{\mathrm{inner}}}\right). \end{array}$$

Since $1\leq \sum e^{x_{\mathrm{inner}}}<N$ after SafeSoftmax processing, substituting this range into the logarithmic formulation yields the range of $x_{\mathrm{outer}}$ is (−16−lnN,0]. Again, inputs below −16 are clipped to zero, consistent with the inner exponential LUT input.

Thus, both exponentials can share one hardware unit. The proposed design achieves a complete division-free Softmax computation using only one subtractor and a small lookup table. By leveraging log-domain decomposition and the scale-invariance of RMSNorm, it maintains numerical precision while significantly reducing hardware complexity in Algorithm 1.
**Algorithm 1:** Proposed Hardware-Efficient Softmax with Log-Domain Approximation
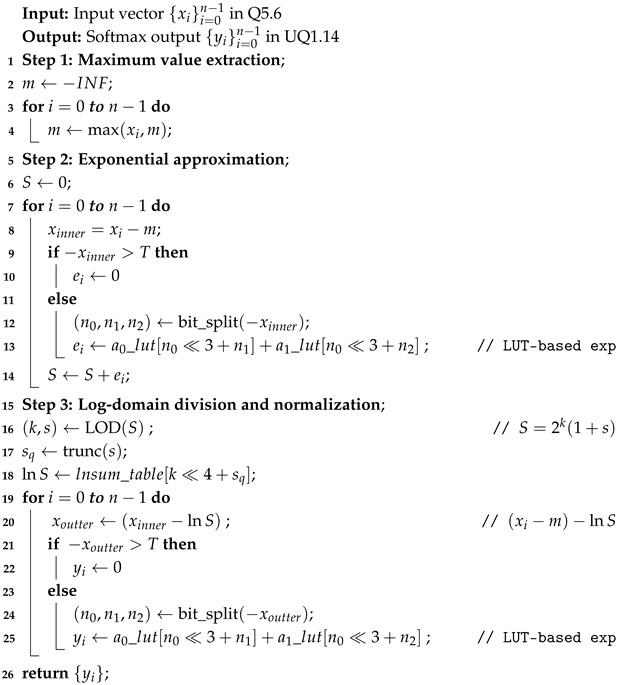


### 4.4. Approximation of Reciprocal Root Computation in RMSNorm

**Figure 4:**
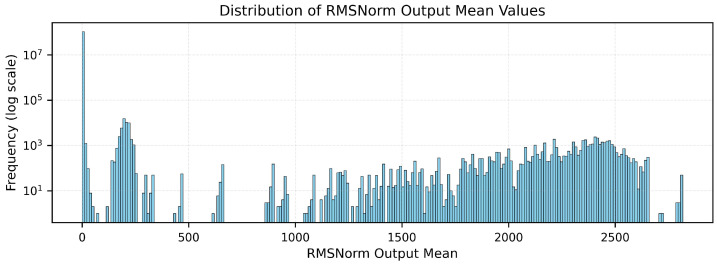
Distribution of the input to the reciprocal square root operation in RMSNorm. The input *d* spans a wide dynamic range, motivating the use of logarithmic leading-one decomposition (LOD) to constrain it into a normalized interval [1,2).

Figure 4 illustrates the statistical distribution of the input to the reciprocal square root operation in RMSNorm, which corresponds to the mean square value *d* computed across all layers. The dynamic range of *d* spans several orders of magnitude, making a direct lookup-based implementation infeasible.

To reduce bit-width and enable efficient table access, the denominator is decomposed using the logarithmic leading-one decomposition (LOD) method as:

(33) $$\frac{1}{\sqrt{d}}=\frac{1}{\sqrt{2^{k}\left(1+s\right)}}=2^{-k/2}\cdot \frac{1}{\sqrt{1+s}},$$

where *k* denotes the position of the leading one in *d*, and s∈[0,1) represents the fractional part after normalization. For example, if $d=110.111_{2}$, then k=2 and $s=0.10111_{2}$. Motivated by [21], the mantissa *s* is truncated to α bits (e.g., α=4), yielding $s=0.1011_{2}$, which is further compensated by a precomputed mean bias to reduce quantization error.

Following the correction approach described in Section 3.3, the truncation error of *s* can be compensated by using a lookup table (LUT) defined as:

(34) $${\mathrm{LUT}}_{s}\left[k_{\alpha }\right]=\frac{1}{\sqrt{1+s}}\approx 2^{\alpha +1}\;\left(\sqrt{1+\left(k_{\alpha }+1\right)2^{-\alpha }}-\sqrt{1+k_{\alpha }2^{-\alpha }}\right),$$

where $k_{\alpha }$ is the integer obtained by truncating *s* to α bits.

Although the exponent term $2^{-k/2}$ seems shift-friendly, its half exponent prevents exact realization through bit shifts. Specifically, when *k* is even, the factor can be computed as a power of two; however, when *k* is odd, it becomes

(35) $$2^{-k/2}=2^{-\lfloor k/2\rfloor }\cdot 2^{-1/2}=2^{-\lfloor k/2\rfloor }/\sqrt{2},$$

which introduces an additional scaling factor of $\sqrt{2}$. Consequently, the baseline design must distinguish between even and odd *k* values, requiring two separate lookup tables (for $1/\sqrt{1+s}$ and $1/\sqrt{2(1+s)}$) and four conditional branches to select the appropriate case, which increases both control complexity and hardware cost.

To eliminate these redundant operations, a unified approach is proposed. Instead of applying shift-based logic and parity checks, we precompute $2^{-k/2}$ directly using a single small LUT indexed by *k*, thereby implicitly covering both even and odd cases without explicit multiplication by $1/\sqrt{2}$. Since the range of *d* in RMSNorm is bounded, a 5-bit representation (k∈[0,31]) is sufficient, requiring only a 32-entry table for the exponent term. Combining the two lookup stages, the complete reciprocal root operation can be expressed as:

(36) $$\frac{1}{\sqrt{d}}=2^{-k/2}\cdot \frac{1}{\sqrt{1+s}}\approx {\mathrm{LUT}}_{k}\left[k\right]\cdot {\mathrm{LUT}}_{s}\left[k_{\alpha }\right].$$

The proposed design minimizes control complexity and memory consumption, utilizing only two compact LUTs together with an integer multiplier: a 32-entry table for the exponent term and a 256-entry table for the mantissa term. The resulting reciprocal root module achieves high precision with minimal hardware overhead, laying the foundation for the proposed hardware-efficient RMSNorm operator described in Algorithm 2.
**Algorithm 2:** RMSNorm with Separate Lookup Tables
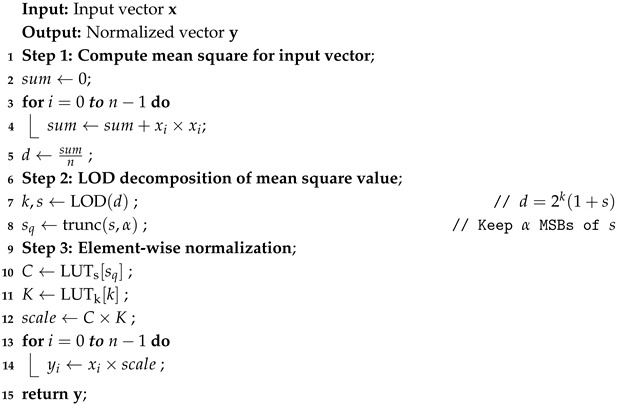


As shown in Table 4, we evaluate the approximation accuracy on 500,000 uniformly sampled points from [0, 2048]. Our Hybrid-LUT approach significantly outperforms the Double-LUT baseline [21] in both accuracy and storage efficiency. The compensated version (Hybrid-LUT*) reduces MAE by 82.4% compared to the compensated Double-LUT* while using 41.3% less storage. Although the improvement from error compensation alone is limited, it still shows certain effectiveness. All configurations maintain similar PPL scores, confirming their practical viability for language modeling tasks.

## 5. Pipelined Implementation of Softmax and RMSNorm Operators

Both Softmax and RMSNorm adopt a unified three-stage pipeline to reduce latency and resource usage. Since we use group-wise fixed-point quantization, we add a fractional-bit alignment unit to unify scaling across groups. Each stage maps to one algorithm phase, enabling high utilization and low dependency.

**Figure 5:**
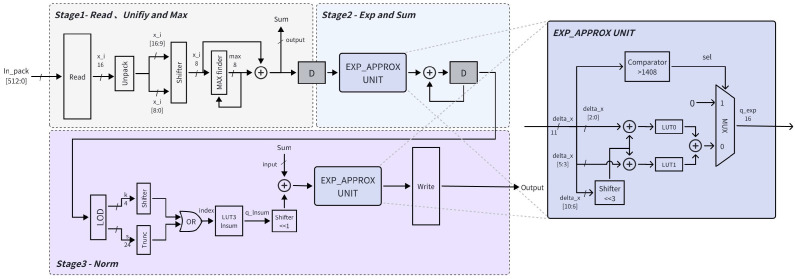
Hardware architecture of the proposed three-stage Softmax pipeline, including input unpacking, exponential computation, and logarithmic-domain normalization.

The complete hardware flow of Softmax is illustrated in Figure 5. In the first stage of the Softmax pipeline, each input value is concatenated with its quantization parameter, namely the fractional-bit position, into a 16-bit short format. Multiple elements are then packed into a 512-bit data block and transmitted to the Softmax processing unit to fully utilize the available bus bandwidth. On-chip, the packed data are unpacked to restore both the input values and their quantization parameters. During this traversal, all elements along the normalization dimension are shifted so that their binary fractional points are aligned to the same position. After alignment, a maximum value search is performed immediately, corresponding to the first loop in the algorithm. After completing *n* cycles, the detected maximum value is forwarded to the next pipeline stage.

In the second stage, each input $x_{i}$ is processed to compute $e^{x_{i}-\max\left(x\right)}$, followed by accumulation to obtain the sum. Prior to exponential evaluation, inputs exceeding the lookup table (LUT) index range are clipped to prevent overflow. This mechanism can also be exploited to mask invalid elements by setting their values and quantization parameters to large numbers, ensuring their exponential outputs clipped to zero. For the 11-bit input, the lower three bits and upper five bits are used as indices for $LUT_{0}$, while the lower three bits and upper five bits index $LUT_{1}$. The outputs from both lookup tables are summed to generate the exponential value, which is then accumulated across all elements. After another *n*-cycle accumulation, the resulting sum is passed to the final pipeline stage.

In the third stage, the leading-one detection (LOD) method is employed to decompose the accumulated sum for logarithmic-domain computation. The leading-one position *k* and mantissa *s* are extracted and concatenated using logic gates to form the final $LUT_{3}$ index. A subsequent fixed bit-shift operation ensures that the computed q_lnsum is aligned with the fractional point of the original sum before accumulation. Finally, the exponential computation unit developed in the second stage is reused in the output stage, achieving efficient hardware reuse and reduced resource overhead.

**Figure 6:**
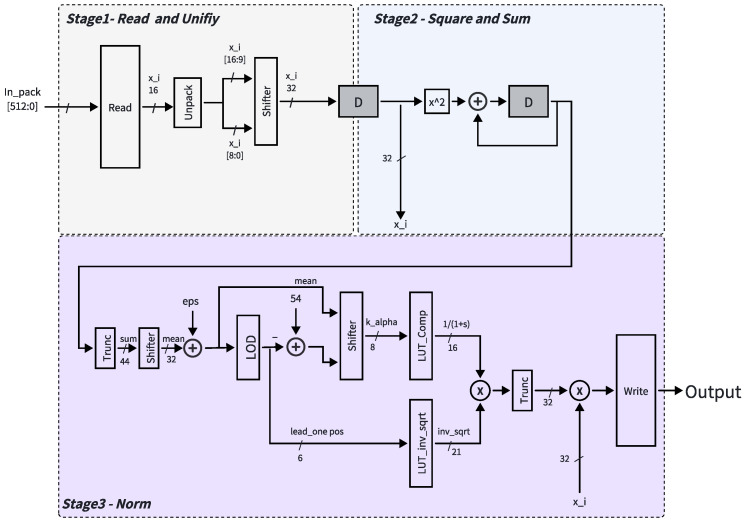
Hardware pipeline of the RMSNorm operator. The overall structure follows the same three-stage design as Softmax, with specific modifications in the squaring, accumulation, and inverse square-root computation stages.

For the RMSNorm operator, the overall pipeline structure remains the same, with differences only in the operations performed in the second and third stages. The complete hardware flow is illustrated in Figure 6. The first stage follows the same unpacking and fractional-bit alignment procedure as Softmax, where the 16-bit short is decomposed into an 8-bit activation value and its corresponding 8-bit quantization parameter. After alignment, each preprocessed input is streamed to the second stage, which performs squaring and accumulation to compute the mean-square value. After *n* cycles, the accumulated result is passed to the third stage, where a fixed bit-shift division is applied to obtain the mean value. An LOD-based decomposition is then performed to extract the leading-one position *k* and the truncated mantissa *s*, which serve as indices for two lookup tables: LUT_inv_sqrt (dependent on *k*) and LUT_comp (dependent on *s*). The corresponding lookup results are multiplied by two integer multipliers to generate the final normalized output. This design requires only two compact lookup tables and two integer multipliers, achieving high numerical precision with minimal hardware cost while preserving a unified pipelined control structure.

## 6. Experiment Results

### 6.1. Experimental Environment

The proposed approximation algorithms for Softmax and RMSNorm were integrated into the LLaMA2-7B model. They were deployed on the AMD Alveo U55C (Advanced Micro Devices, Inc., Santa Clara, CA, USA) accelerator card to evaluate hardware resource usage, performance, and energy efficiency. To ensure that the proposed approximations do not introduce unacceptable numerical degradation, model accuracy was evaluated on the Wikitext dataset under identical experimental conditions. In addition, a non-functional requirement (NFR) classification dataset for industrial software development scenarios was used to evaluate the proposed methods in a downstream application task.

Software-level simulation of the proposed approximation algorithms was conducted using the PyTorch framework, while heterogeneous inference was executed through the llama.cpp runtime. The heterogeneous computing platform consisted of an AMD Ryzen 9 7950X (Advanced Micro Devices, Inc., Santa Clara, CA, USA) host processor and a AMD Alveo U55C accelerator card, operating under Ubuntu 20.04.3 LTS (Canonical Group Limited, London, UK) The hardware acceleration circuits were implemented with the Vitis HLS 2022.1 (Advanced Micro Devices, Inc., Santa Clara, CA, USA) toolchain, and bitstreams were generated using Vivado 2022.1 (Advanced Micro Devices, Inc., Santa Clara, CA, USA). The generated bitstream was loaded to the U55C accelerator via a PCIe interface. The accelerator provides abundant on-chip resources, including approximately $1.304\;\times \;10^{6}$ lookup tables (LUTs), 9024 DSP slices, 70.9 Mbit of BRAM, and 16 GB of high-bandwidth memory (HBM), enabling high-throughput computation for transformer-based model inference.

### 6.2. Datasets

Two datasets were used in this study for evaluating both language modeling accuracy and downstream software engineering requirement classification tasks. The Wikitext-2 dataset [29] is a language modeling corpus constructed from high-quality English Wikipedia articles. It preserves the original document structure while removing machine-generated content. Wikitext-2 is employed as a benchmark for evaluating model perplexity (PPL) and assessing text prediction capability.

The PROMISE-relabeled-NICE dataset, on the other hand, is a benchmark widely used in the software engineering domain for non-functional requirement (NFR) identification and classification. It originates from early studies by Cleland-Huang et al., who manually annotated requirement documents for evaluating automated NFR detection and classification methods. It is later re-labeled by Dalpiaz et al., introducing a multi-label scheme that allows each requirement to simultaneously exhibit F (functional aspects), Q (quality aspects), OnlyF (only functional aspects), and OnlyQ (only quality aspects) attributes. The NFR subset is further extended in the NICE approach [30] into 12 subcategories, and the final dataset contains 622 labeled instances. This dataset is used to evaluate the model performance in the downstream requirement classification task.

### 6.3. Software Engineering Requirement Classification

**Figure 7:**
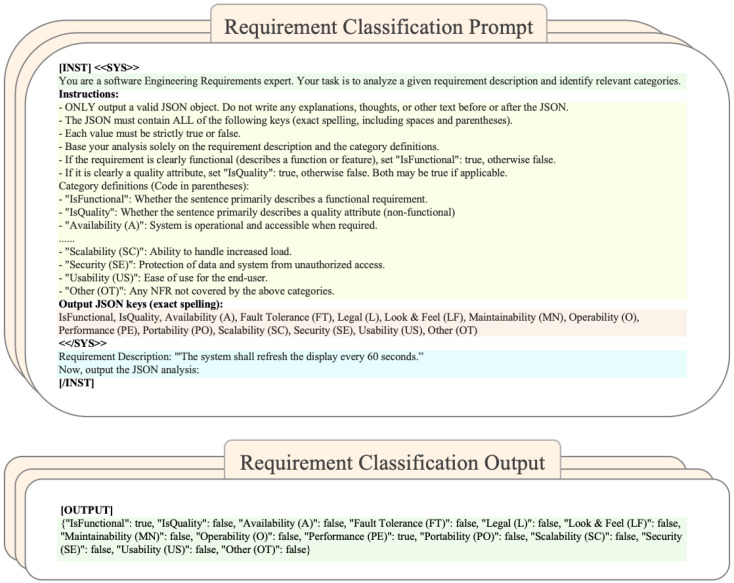
Illustration of the requirement classification prompt and output format. The upper part shows the designed instruction prompt used for model inference, and the lower part presents the corresponding structured JSON output.

To perform multi-label requirement classification in the software engineering domain, a general-purpose prompt was constructed to inform the model of the task background, detailed instructions, required output format, and the concatenated requirement description. The overall structure of the prompt and corresponding output format are illustrated in Figure 7, which defines the instruction template, output schema, and example of requirement-to-label mapping.

The model is quantized to W8A8 precision. To preserve perplexity performance, group-wise quantization is applied to the inputs of both the Softmax and RMSNorm operators, and the quantized model is deployed on the Alveo U55C hardware platform based on the llama.cpp framework. Each input prompt containes approximately 550 tokens, and the corresponding output classification information consisted of about 106 tokens. The measured average latency from receiving a requirement text to generating the complete JSON label output is approximately 6 s per instance. This level of latency is suitable for offline requirement analysis and decision-support scenarios. For requirement engineering practices where analysts determine requirement categories interactively for each item, such latency remains acceptable. The model achieves a precision of 0.7769, a macro recall of 0.6533, and a macro F1 score of 0.7097, as shown in Table 5. Importantly, these results are obtained with the temperature parameter set to zero (‘temp = 0’), which eliminates sampling randomness and ensures completely deterministic predictions. The approximation shows selective impact across categories: while overall performance degradation is minimal ($\Delta_{macro}=-0.016$), Portability (PO) and Fault Tolerance (FT) experience notable declines, whereas Scalability (SC) and Availability (A) show improvement. The core IsFunctional category remains unaffected. This demonstrates engineering applicability with respect to inference latency. Furthermore, additional optimization opportunities remain through prompt compression and output simplification, which could further reduce overall response time and enable real-time or high-concurrency industrial deployment.

### 6.4. Analysis and Comparison

Table 6 reports the perplexity (PPL) of the proposed Softmax and RMSNorm approximation methods under different quantization and deployment settings on the Wikitext dataset. The FP16 configuration serves as the baseline, while the W8A8 setup introduces quantization and operator approximations stepwise to assess their impact on model accuracy.

Under FP16 precision, PyTorch and CPU simulation yield nearly identical PPLs (5.4762 vs. 5.4736), confirming that platform differences do not affect model behavior in the absence of quantization or approximation. Across all configurations, the discrepancy between CPU simulation and FPGA deployment remains below 0.002, confirming that our design precisely reproduces the software model. Based on the CPU results in Table 6, the total perplexity increase from the FP16 baseline (PPL = 5.4736) to the configuration with both Softmax and RMSNorm approximations (PPL = 5.4973) is 0.0237. Among this increase, W8A8 quantization alone contributes 0.0204 (from 5.4736 to 5.4940), accounting for approximately 86% of the total, while the combined Softmax and RMSNorm approximations contribute the remaining 14%.

To explicitly quantify the impact of individual nonlinear operator approximations under quantization, we conduct an operator-level ablation study on Wikitext-2 under W8A8 group-wise quantization (group size = 32). In this study, Softmax and RMSNorm are approximated independently while keeping all other components unchanged, allowing us to isolate the contribution of each approximation to the overall perplexity degradation.

As shown in Table 7, both Softmax and RMSNorm approximations introduce only marginal additional perplexity degradation beyond quantization. Among the evaluated components, Softmax exhibits slightly higher sensitivity to approximation, especially when log-domain division is applied, due to the exponential and normalization operations involved. In contrast, RMSNorm with the hybrid LUT-based reciprocal square root introducing only +0.0015. These results confirm that the proposed operator-level approximations are well-balanced.

The hardware comparisons in Table 8 and Table 9 are based on resource utilization results reported in the original publications. The FPGA platforms used in the compared works are mainstream Xilinx devices that share a common programmable logic abstraction, including LUT-based logic fabric and DSP slices, enabling meaningful operator-level comparison. To improve fairness, we additionally report the resource utilization ratio (%) on our target device. Although different FPGA devices and toolchains may lead to variations in absolute resource usage, such variations are generally limited and do not affect the overall trends reflected in our results. Given the very low utilization levels of the proposed operators, the impact of cross-platform differences is further reduced, making operator-level qualitative comparison across different FPGA platforms reasonable.

The hardware comparisons in Table 8 and Table 9 are based on results reported in prior works. The compared designs are implemented on different FPGA devices that belong to the same generation and fabrication process. Differences among these devices mainly lie in available resource capacity, rather than architectural differences. Additionally, since the proposed Softmax and RMSNorm operators occupy less than 5% of on-chip resources, and all compared designs are implemented using the Vivado toolchain, the impact of placement-and-routing variability is negligible.

Table 8 compares the hardware resource utilization of the proposed Softmax implementation with several representative prior works. Compared with previous designs, the proposed implementation achieves the lowest DSP usage and significantly better LUT efficiency. Specifically, on the Xilinx Alveo U55C platform, the design operates at 300 MHz and consumes only 3112 LUTs and 6215 registers, representing an 83% reduction in LUT utilization compared with [31], while maintaining comparable register usage to lightweight implementations such as [32]. Furthermore, by employing a log-domain transformation and a Bi-lut-comp scheme to replace exponential and division operations, the proposed design requires zero DSPs, whereas [33] rely on up to 128 DSPs for high-precision exponentiation. Although the method uses 10.5 BRAMs for table storage, this overhead remains substantially lower than the 72 KB memory reported in [34] for floating-point computation. These results demonstrate that the proposed Softmax achieves superior hardware efficiency and is highly suitable for low-power or resource-constrained FPGA deployment scenarios.

**Table 8:** Resource utilization comparison of the proposed Softmax implementation with representative prior works.

FPGA Device	Ours	Ref. [31]	Ref. [35]	Ref. [33]	Ref. [19]	Ref. [34]	Ref. [32]
Type	Xilinx Alveo U55C	Xilinx Zynq	Xilinx Kintex-7 KC705	Xilinx ZCU102	Xilinx XCVU13P	Xilinx Virtex6 ML605	Xilinx Zynq-7000 ZC706
Freq. (MHz)	300	150	154	300	200	400	294
LUT	3112 (0.239%)	17,870	2229	22,865	21,190	300	1858
Registers	6215 (0.238%)	16,400	224	21,770	32,623	558	2086
DSP	0 (0%)	–	–	128	0	5	8
BRAM	10.5 (0.530%)	–	–	0	0	72K	–

Table 9 compares the hardware resource utilization of the proposed RMSNorm implementation with several representative prior works. Our implementation operates at 300 MHz while utilizing only 3954 LUTs and 5810 registers—substantially fewer than the 10k+ LUT designs reported in [19,33], and over 95% fewer logic resources compared with [22]. Moreover, the design consumes only 18 DSPs for reciprocal square root approximation, significantly less than the 74, 129, and 1025 DSPs required in [19,22,33] for floating-point computation, underscoring its hardware-friendly nature. In terms of memory usage, 16 BRAMs are employed for lookup-table storage—slightly higher than some integer-based implementations, yet far below the 27.5 BRAMs required for CORDIC iterations in [19]. Overall, the proposed RMSNorm achieves high numerical precision with minimal hardware overhead, making it well-suited for efficient FPGA deployment.

To provide a clearer system-level perspective, we analyze the resource utilization and scalability of the proposed Softmax and RMSNorm operators on the AMD Alveo U55C FPGA. As reported in Table 8 a single Softmax instance consumes only 0.239% of LUTs, 0.238% of registers, 0% of DSPs, and 0.53% of BRAM. Similarly, a single RMSNorm instance occupies 0.303% of LUTs, 0.223% of registers, 0.199% of DSPs, and 0.81% of BRAM as shown in Table 9. When deployed together as a paired Softmax-RMSNorm pipeline, the combined resource usage remains small, amounting to 0.54% of LUTs, 0.46% of registers, 0.20% of DSPs, and 1.35% of BRAM. Such a low per-instance footprint allows the proposed operators to be replicated many times on a single device. Based on available on-chip resources, up to approximately 192 Softmax instances, 126 RMSNorm instances, or 76 paired Softmax–RMSNorm pipelines could theoretically be instantiated in parallel on U55C. In all cases, BRAM capacity becomes the first limiting resource, while LUT, register, and DSP utilization remains well below saturation. This analysis indicates that the proposed operators are lightweight, highly scalable, and unlikely to form a bottleneck in a full Transformer inference pipeline.

**Table 9:** Resource utilization comparison of the proposed RMSNorm implementation with representative prior works.

FPGA Device	Ours	Ref. [33]	Ref. [19]	Ref. [22]	Ref. [36]
Type	Xilinx Alveo U55C	Xilinx ZCU102	Xilinx XCVU13P	Xilinx U280	Xilinx Alveo U50
Freq. (MHz)	300	300	200	100	100
LUT	3954 (0.303%)	10,558	10,551	86K	2817
Registers	5810 (0.223%)	4038	5325	25K	2145
DSP	18 (0.199%)	74	129	1025	7
BRAM	16 (0.810%)	9	27.5	–	2

The proposed Softmax and RMSNorm operators are integrated into the standard Transformer inference pipeline, where Softmax is applied within the self-attention module and RMSNorm is used both before and after attention along the residual paths. In our implementation, activations are first grouped and quantized to 8-bit precision, after which all Softmax and RMSNorm computations are carried out using the proposed fixed-point approximation modules. Although these operators account for fewer FLOPs than attention matrix multiplications and MLP layers, they are executed for every layer and every token and therefore lie on the critical inference path.

To evaluate the practical performance of the proposed operators, we report the measured execution latency and power consumption of the FPGA implementation under representative workload configurations. Power consumption is estimated using the Vivado Report Power utility on the post-implementation design at 300 MHz. For Softmax with a data size of 32×2048, the FPGA implementation achieves a latency of 0.292 ms at 300 MHz, with a power consumption of 17.7 W. This corresponds to an effective throughput of 224.4 M elements/s. For RMSNorm with a data size of 128×4096, the measured latency is 1.837 ms, while consuming 17.6 W. The corresponding throughput is 285.4 M elements/s. These results reflect the operator-level execution cost of the proposed pipelined designs on FPGA and complement the resource and scalability analysis discussed above.

## 7. Conclusions

This paper presented a hardware-efficient approximation and accelerator framework for the Softmax and RMSNorm operators, targeting transformer inference acceleration on FPGA platforms. By combining range-reduced bipartite lookup tables with bit-width optimization, the proposed Softmax achieves high numerical accuracy while eliminating costly division operations through log-domain reformulation. For RMSNorm, a leading-one-based logarithmic decomposition and Hybrid LUT-based reciprocal–root approximation effectively replace floating-point arithmetic with integer operations, preserving precision and simplifying control logic.

Experimental results show that, when integrated into the LLaMA2-7B model, the proposed designs maintain nearly identical accuracy to the FP16 baseline, with only 0.026 perplexity deviation on Wikitext-2. On the Xilinx Alveo U55C, the Softmax and RMSNorm modules run at 300 MHz while consuming only 3112 and 3954 LUTs, respectively, and requiring merely 0 and 18 DSPs—achieving substantial resource savings. Both operators run at 300 MHz with low operator-level latency and modest power consumption, while requiring only a small fraction of on-chip logic and DSP resources.

Overall, the proposed approximation and accelerator achieve a favorable balance between precision, efficiency, and generality, demonstrating strong potential for low-power transformer inference and other resource-constrained AI deployments on FPGA platforms.

## Figures and Tables

**Table 1:** Different configurations of input and output fractional bitwidths.

Input Fraction Bitwidth	Output Fraction Bitwidth	PPL
6	14	5.5013
6	15	5.5002
7	14	5.4993
7	15	5.4940

**Table 2:** Error experiments under different bit-width configuration.

Configuration $\left(n_{0},n_{1},n_{2}\right)$	MSE	MAE	MAX	Total Storage (bits)	PPL
(5, 3, 3)	0.00002339	0.00357613	0.01893544	512 × 16 (8192)	5.4955±0.02154
(5, 4, 2)	0.00000307	0.00141050	0.00530612	640 × 16 (10,240)	5.4948±0.02150
(6, 3, 2)	0.00000239	0.00126817	0.00463378	768 × 16 (12,288)	5.4944±0.02149

**Table 3:** Accuracy and storage requirements comparison under (5, 3, 3) configuration.

Method	MSE	MAE	MAX	Total Storage (bit)	PPL
Naive-lut	0.00000220	0.00123727	0.00389008	2048 × 15 (30,720)	5.4943 ± 0.02146
Bi-lut-uncomp	0.00002339	0.00357613	0.01893544	512 × 15 (7680)	5.4949 ± 0.02157
Bi-lut-comp (ours)	0.00000510	0.00176482	0.00784302	512 × 15 (7680)	5.4946 ± 0.02147

**Table 4:** Accuracy and storage comparison for $1/\sqrt{d}$ approximation.

Method	RMSE ($\times 10^{-3}$)	MAE ($\times 10^{-3}$)	MAX ($\times 10^{-3}$)	Total Storage (bit)	PPL
Double-LUT [21]	74.600	23.475	362.39	256×16+256×16 (8192)	5.5010±0.02144
Double-LUT * [21]	72.082	22.305	362.74	256×16+256×16 (8192)	5.5008±0.02157
Hybrid-LUT (ours)	18.005	3.9321	187.88	256×16+34×21 (4810)	5.4956±0.02148
Hybrid-LUT * (ours)	17.921	3.9184	186.88	256×16+34×21 (4810)	5.4955±0.02147

* Methods with asterisk indicate versions with error compensation.

**Table 5:** Performance Comparison of NFR Classification Before and After Model Approximation (F1-Score).

Requirement Category (Label)	After Approximation	Original FP16 Model	Difference (Δ)
IsFunctional	0.88	0.88	0.00
Availability (A)	0.86	0.83	+0.03
Look & Feel (LF)	0.87	0.85	+0.02
Scalability (SC)	0.86	0.79	+0.07
Legal (L)	0.83	0.81	+0.02
Usability (US)	0.77	0.73	+0.04
Security (SE)	0.72	0.76	−0.04
IsQuality	0.72	0.70	+0.02
Performance (PE)	0.70	0.67	+0.03
Fault Tolerance (FT)	0.67	0.78	−0.11
Portability (PO)	0.51	0.71	−0.20
Maintainability (MN)	0.45	0.48	−0.03
Operability (O)	0.40	0.45	−0.05
Macro Average	0.710	0.726	−0.016

**Table 6:** Accuracy comparison of Softmax and RMSNorm approximation methods across platforms on the Wikitext dataset. Here, “CPU” denotes the software simulation environment implemented in the llama.cpp framework, used to verify the numerical equivalence of the proposed approximation before FPGA deployment.

Quantization	Configuration	PyTorch	CPU	CPU+FPGA	PPL
FP16	–	✓	✗	✗	5.4762
	–	✗	✓	✗	5.4736
	–	✓	✗	✗	5.4825
W8A8	–	✗	✓	✗	5.4940
		✓	✗	✗	5.4799
	+Softmax_Approx	✗	✓	✗	5.4971
		✗	✗	✓	5.4984
		✓	✗	✗	5.4792
	+RMSNorm_Approx	✗	✓	✗	5.4955
		✗	✗	✓	5.4967
		✓	✗	✗	5.4821
	+Softmax_A+RMSNorm_A	✗	✓	✗	5.4973
		✗	✗	✓	5.4992

**Table 7:** Operator-level ablation of Softmax and RMSNorm approximations under W8A8 group-wise quantization (group size = 32) on CPU.

Component	Approximation Variant	PPL	ΔPPL
Baseline	Quantization only (no approximation)	5.4940	–
Softmax	LUT-based exp + standard division	5.4951	+0.0011
	LUT-based exp + log-domain division (ours)	5.4971	+0.0021
RMSNorm	standard division	5.4958	+0.0018
	Hybrid LUT-based division (ours)	5.4955	+0.0015

## Data Availability

The original contributions presented in the study are included in the article, further inquiries can be directed to the corresponding author.
